# Dissolving the Psychological Subject: Inside and Outside the Therapeutic Bond

**DOI:** 10.1111/1468-5922.13089

**Published:** 2025-04-08

**Authors:** Mark Saban

**Affiliations:** ^1^ Oxford UK

**Keywords:** affect, emotion, interactive field, Jung, Simondon, the third, transindividuation, Jung, affect, émotion, Simondon, transindividuation, champ interactif, troisième champ, Jung, Affekt, Emotion, Simondon, Transindividuation, interaktives Feld, das Dritte, Jung, affetti, emozioni, Simondon, transindividuazione, campo interattivo, il terzo, Юнг, аффект, эмоция, Симондон, трансиндивидуация, интерактивное поле, третье, Jung, afecto, emoción, Simondon, transindividuación, campo interactivo, el tercero, 荣格, 情感, 情绪, 西蒙东, 超个体化, 互动场域, 第三

## Abstract

This paper focuses on the role of affect within the process of individuation. This approach provides us with the opportunity to shed light upon the (mostly implicit) capacity Jung’s psychology possesses to move beyond the limitations of individuality and to highlight the psyche’s collective dimension. The paper explores some of the ways in which we can become aware of such processes within the vessel of clinical work. A recap of Jung’s comments on affect and emotion is followed by a discussion of the contribution of the French philosopher Gilbert Simondon and particularly the role of affect in what he calls “transindividuation” (Simondon, 2020; Combes, 2013). Clinical examples will be provided to illustrate the way in which powerful affects can be observed to occur not only within the separate psyches of either patient or analyst, but also within a third field or “bond” (Jung, 1946, para. 367) which I believe we can usefully understand as “transindividual”.

## Abstract (fr)

Cet article se concentre sur le rôle de l'affect dans le processus d'individuation. Cette approche nous permet de mettre en lumière la capacité (le plus souvent implicite) de la psychologie de Jung à dépasser les limites de l'individualité et à mettre en évidence la dimension collective de la psyché. L'article explore quelques‐unes des façons dont nous pouvons prendre conscience de ces processus dans le cadre du travail clinique. Un rappel des commentaires de Jung sur l'affect et l'émotion est suivi d'une discussion sur la contribution du philosophe français Gilbert Simondon et en particulier sur le rôle de l'affect dans ce qu'il appelle la « transindividuation » (Simondon, 2020; Combes, 2013). Des exemples cliniques sont amenés pour illustrer la façon dont on peut observer que des affects puissants se produisent non seulement dans les psychés séparées du patient ou de l'analyste, mais aussi dans un troisième champ ou « lien » (Jung, 1946, par. 367) qui, je crois, peut être utilement compris comme « transindividuel ».

## Abstract (de)

In diesem Artikel wird die Rolle der Affekte im Prozeß der Individuation untersucht. Dieser Ansatz bietet uns die Möglichkeit, die (meist implizite) Fähigkeit von Jungs Psychologie zu beleuchten, über die Grenzen der Individualität hinauszugehen und die kollektive Dimension der Psyche hervorzuheben. Der Beitrag untersucht einige der Möglichkeiten, wie wir uns im Rahmen der klinischen Arbeit solcher Prozesse bewußt werden können. Auf eine Zusammenfassung von Jungs Kommentaren zu Affekt und Emotion folgt eine Diskussion über den Beitrag des französischen Philosophen Gilbert Simondon und insbesondere die Rolle des Affekts bei dem, was er “Transindividuation” nennt (Simondon 2020, Combes, 2013). Anhand klinischer Beispiele soll veranschaulicht werden, wie starke Affekte nicht nur in der jeweiligen Psyche des Patienten und des Analytikers auftreten können, sondern auch in einem dritten Feld oder einer “Bindung” (Jung, 1946, Abs. 367), die wir meines Erachtens sinnvollerweise als “transindividuell” betrachten können.

## Abstract (it)

Il presente articolo tratta del ruolo degli affetti nel processo di individuazione. Questo approccio ci offre l'opportunità di gettare una luce sulle potenzialità (perlopiù implicite) che la psicologia junghiana possiede per superare i limiti dell'individualità e vedere la dimensione della psiche collettiva. Vengono esplorati alcuni dei modi in cui noi riusciamo ad avere consapevolezza di questi processi, all'interno del lavoro analitico. Ad un breve riepilogo delle affermazioni di Jung sugli affetti e sulle emozioni seguono le riflessioni sul contributo del filosofo francese Gilbert Simondon e, più in particolare, sul ruolo degli affetti per ciò che lui chiama “transindividuazione” (Simondon, 2020; Combes, 2013). Vengono presentati esempi clinici che mostrano in che modo gli affetti potenti possono essere osservati in azione, non solo all'interno della psiche del paziente ed in quella dell'analista, ma all'interno di un terzo campo o “legame” (Jung, 1946, para. 367) che, credo, possiamo considerare come “transindividuale”.

## Abstract (ru)

Статья посвящена роли аффекта в процессе индивидуации. Этот подход помогает пролить свет на (предполагаемую) способность психологии Юнга выйти за пределы индивидуальной личности и увидеть коллективное измерение психики. В статье рассматриваются некоторые способы, с помощью которых мы можем получить представление о таких процессах в рамках клинической работы. После краткого обзора комментариев Юнга об аффекте и эмоциях следует обсуждение концепции французского философа Жильбера Симондона – в частности, его идея о роли аффекта в том, что он называет “трансиндивидуацией” (Simondon, 2020; Combes, 2013). Приведенные клинические примеры иллюстрируют, как можно наблюдать возникновение сильных аффектов не только в психике пациента или аналитика по отдельности, но и в третьем поле, или «связи» (Юнг, 1946, пункт 367), которую, я полагаю, можно назвать «трансиндивидуальной».

## Abstract (es)

Este artículo se centra en el papel de las emociones en el proceso de individuación. Este enfoque nos brinda la oportunidad de arrojar luz sobre la capacidad (en su mayor parte implícita) que posee la psicología de Jung para ir más allá de las limitaciones de la individualidad y poner de relieve la dimensión colectiva de la psique. El artículo explora algunas de las formas en que podemos tomar conciencia de tales procesos dentro del contenedor del trabajo clínico. Tras una recapitulación de los comentarios de Jung sobre el afecto y la emoción, se analiza la contribución del filósofo francés Gilbert Simondon y, en particular, el papel del afecto en lo que él denomina «transindividuación» (Simondon, 2020; Combes, 2013). Se proporcionarán ejemplos clínicos para ilustrar la forma en la que poderosas emociones tienen lugar no sólo en las psiques de paciente o analista respectivamente, sino también dentro de un tercer campo o «vínculo» (Jung, 1946, párr. 367) que, creo, podemos entender útilmente como «transindividual».

## Abstract (zh)

溶解心理的主体:在治疗关系内外

本文聚焦于情感在自性化过程中的作用。这种方法为我们提供了一个机会, 让我们能够揭示荣格心理学 (多数时候深藏不露的)能力, 即超越个体性局限的能力, 以及对心理的集体维度的凸显。文章探讨了一些方法, 让我们可以在临床工作的容器中意识到这些过程。文章回顾了荣格对情感和情绪的评论, 随后讨论了法国哲学家吉尔伯特·西蒙东的贡献, 特别是情感在他所说的“超个体化”中的作用 (Simondon, 2020; Combes, 2013)。本文将提供临床案例, 以说明强大的情感不仅可以在患者或分析师的各自独立的心理中被观察到, 还可以在一个第三个场域, 或称之为“联结”(荣格, 1946, 第367段)中被观察到, 我相信, 我们可以将这理解为“超个体”。

In this paper, I intend to focus on the role of affect within the process of individuation and thus to shed light upon the (mostly implicit) capacity Jung’s psychology possesses to put aside the limitations of individuality and to highlight the collective dimension of the psyche.

After briefly recapping what Jung has to say about affect and emotion, I shall move on to discuss the contribution of the French philosopher Gilbert Simondon and particularly the role of affect in the process he describes as “transindividuation” (Simondon, 2020, Combes, 2013). I will then give some clinical examples to illustrate the way in which powerful affects show up not only within the separate psyches of either patient or analyst, but also within a third field or “bond” (Jung, 1946, para. 367) which, I believe we can usefully understand as ‘transindividual’.

Any contemporary discussion of emotion or affect needs to take into account the so‐called “turn to affect” (Brennan, 2004), a recent transdisciplinary shift within the humanities and social sciences that has prioritized the role and importance of affectivity. The influence of this “affective turn” ranges across the fields of Human geography, philosophy, psychology, sociology and body studies (Blackman, 2012; Brennan, 2004; Clough, 2007; Massumi, 2015; Pile, 2010; Slaby & von Scheve, 2019; Wetherell, 2012). In brief, this approach highlights the autonomy of affective relations and their capacity to challenge the notion of the bounded individual.

In recent years, there has also been an increased interest in affect within analytical psychology and psychoanalysis, much of which has taken a neuroscientific form. (e.g., Alcaro, Carta & Panksepp, 2017) Despite the undoubted value of this research, (particularly in its tendency to highlight human affect’s continuity with the non‐human world), it nonetheless tends to be presented within a conventional scientific discourse which, by highlighting the individual psyche (or brain), thus implicitly supports precisely the Cartesian approach that the turn to affect seeks to challenge.

It should be noted that for the purposes of my argument I will distinguish the concept of affect from that of emotion. Affect should be thought of as a collective, impersonal and indeed interpersonal phenomenon and emotion as an intrapsychic, private and personal phenomenon. In effect, affect is to emotion what the collective unconscious is to the personal unconscious. To paraphrase Jung, we have emotions but affects have us.

## Jung and Affect

Affect‐emotion (Jung doesn’t distinguish between the two) appears to have played an important part in the early development of Jung’s psychological understanding. In the period of his collaboration with Freud, Jung regarded affect as “the binding force of the psychic organism” (Vandermeersch, 1991, p. 109). As he clearly states, “The essential basis of our personality is affectivity. Thought and action are, as it were, only symptoms of affectivity” (Jung, 1907, para. 78). Note that this notion differs radically from Freud’s understanding of affect as a second‐order product of instinctive energies. 1

The importance of affect also shows up in Jung’s early writings on the so‐called feeling‐toned complex. As Roger Brooke puts it, Jung sees complexes as “centres of affective gravity… [that give] density, shape, and limitation to consciousness. Consciousness [is] both supported and skewed by an affective power that could not be accounted for in terms of consciousness alone” (Brooke, 1991, p. 507).

These complexes—affective nodes or ‘fragmentary psychic systems’—strongly inflected those images of inner persons that Jung was to meet and relate to during his ‘confrontation with the unconscious’, experiences that, as Jung acknowledges, were saturated in affect (Jung & Jaffé, 1989, p. 177). Indeed, he goes on to develop the notion that it is precisely affective events of this kind—usually in the form of an “unwelcome intrusion from the unconscious” (Jung, 1916, para. 167)—that invariably constitute the starting point—the spark—for creative engagement between conscious ego and the deep unconscious.

Later, as he develops his mature psychology, Jung repeatedly highlights the fact that our encounters with the unconscious—the unknown other—show up in the form of affective events. 2 The importance of affect derives from its capacity to open up a relational link between ego and other. In a sense, affective experiences enable the unconscious to grab the ego’s attention. Affect—whether it is love, fear or hate—breaks up the defences of the ego by forcing it out of its comfort zone.

In short, the destabilizing experience of affect is what characterizes our confrontations with the other—whether it be the outer other—I hate this place, I fear this animal, I love this man—or the inner other—i.e., those phenomena that we characterize as unconscious. No matter where or when it occurs, the experience of affect is, in Jung’s words, “as if an explosion had moved you out of yourself and put you beside yourself” (Jung, 1935, para. 46). Affect is therefore always a threat to the psychic status quo because it signals and expresses a violent influx of the other.

For example, we know that a particular dream matters and needs attention because we awake disturbed (affected) by it. In other words, the ego can’t find a way to sideline or diminish the sadness, fear or desire that has arisen. The more transgressive and disruptive these events are, Jung says, the greater their psychological significance. Their heightened feeling tone indicates, “the degree to which the subject is affected by the process or how much it means to him… It is through the ‘affect’ that the subject becomes involved and so comes to feel the whole weight of reality” (Jung, 1951, para. 61)

Ultimately, for Jung, affect‐emotion is nothing less than a world‐creating force:

[E]motion is the alchemical fire whose warmth brings everything into existence and whose heat burns all superfluities to ashes… But on the other hand, emotion is the moment when steel meets flint and a spark is struck forth, for emotion is the chief source of consciousness. ( Jung, 1954, para. 179)

## Affect and Archetype

At this point, it is important to note Jung’s insistence upon the intimate relationship between affect‐emotion and archetype. Archetypes, he says “are images and at the same time emotions. One can speak of an archetype only when these two aspects coincide” (Jung, 1961, para. 589). Evidently then, the experience is not merely personal; for Jung, affect puts us in touch with that which transcends the individual. Jung characteristically expresses this in terms of numinosity and the God‐image:

Whenever… in an excess of affect, in an emotionally excessive situation, I come up against a paradoxical fact or happening, I am in the last resort encountering an aspect of God, which I cannot judge logically and cannot conquer because it is stronger than me—because, in other words, it has a numinous quality…I cannot “conquer” a numinosum, I can only open myself to it, let myself be overpowered by it, trusting in its meaning. ( Jung, 1959, para. 864)

## Affect

Many recent writers on affect trace their ideas back to the 17^th^ century Dutch philosopher Spinoza, who describes affect simply as the capacity to affect and to be affected (Massumi, 2015, p. ix). One value of this approach lies in its emphasis upon the fact that when we affect or are affected by other people, or other things, we are in relationship with them, whether they are experienced as inner or outer phenomena. In other words, affect puts us in touch with and highlights wider relational processes. As Brian Massumi puts it, affects are “our angle of participation in processes larger than ourselves. With intensified affect comes a stronger sense of embeddedness in a larger field of life—a heightened sense of belonging, with other people and to other places” (Massumi, 2015, p. 6).

Affect thus possesses the capacity to radically subvert our accustomed style of engagement with the world because it initiates a process of profound de‐centring. Through affect we experience the transitions and thresholds of change in our lives. These mutative affective moments are felt intensively, but through them we gain a sense of expanse, gaining access to a world less limited to our narrow ego‐emotions and ego‐thoughts. Paradoxically, while the intensity of affect is felt personally and subjectively, it simultaneously somehow connects us to a non‐personal, non‐subjective, non‐individual dimension of existence. In Jungian terms, this latter dimension is equivalent to what Jung calls the collective unconscious. In Simondon’s terms, as we will see, it is the pre‐individual.

## Simondon

The ideas of Gilbert Simondon—a French philosopher who lived from 1924 to 1989—have proved particularly helpful when unpicking problems in Jung’s psychology and specifically those questions that relate to the tension between the inner world and the outer world (Saban, 2020). Simondon’s *magnum opus* (only recently fully translated into English) is entitled *Individuation in Light of Notions of Form and Information* (Simondon, 2020). As a philosopher, Simondon extends Jung’s notion of individuation into regions far wider than the psychological. Jung is by no means the only source for his ideas, but Simondon does repeatedly take the opportunity to refer to Jung’s psychology, often comparing it favourably to Freud’s psychoanalysis. The numerous ways in which Simondon’s ideas resonate with and amplify Jung’s psychology exceed the limits of this paper. For now, I wish to highlight a single concept of Simondon: transindividuation.

In order to grasp this notion, we need first to acknowledge the difference between Jung’s approach to individuation and that of Simondon. For Jung, the individuation process is essentially an inner process undertaken and completed within the psychological subject. In individuation, the narrow ego perspective of the individual gets challenged and de‐centred through awareness of the wider Self. However, for Jung all this is an *inner* process. Even Jung’s emphasis upon the psychic collective (as in the “collective unconscious”) tends to be expressed as operating on the inner level. And however much Jung may emphasize—and he does—that the newly individuated individual will, as a consequence of individuation, be better equipped to relate outwardly to the outer collective, this tends to be represented as a secondary gain, brought about by a process that is essentially interior.

Simondon’s notion of the transindividual takes up Jung’s idea and pushes it much further. For Simondon, Jung’s approach is unnecessarily narrow. What Jung calls individuation is, for Simondon, merely the *initial* phase within a more extensive process of psychic individuation. For Simondon individuation culminates in what he calls transindividuation—an operation that he thinks can only take place in relation to the collective.

What Simondon calls the preindividual dimension of the subject closely parallels Jung’s collective unconscious, in that it exceeds and is anterior to the constituted individual. Never exhausted by the individual, the preindividual is carried forward within the individual, such that the constituted individual always contains within itself, *in potentia*, a charge of preindividual reality—a reality animated by, and rich in, potentials. This is all reminiscent of what Jung would call “archetypal” potential. Jung’s collective unconscious too exceeds the narrow ego and, in a sense, remains available to every human subject. For both Jung and Simondon, key events of individuation occur at moments when the subject’s self or ego‐unity gets thrown into doubt or crisis. Both agree that the process of individuation has the effect of resolving these problematic experiences (in which we are made aware of inner disunity) in such a way that a new (and hitherto unpredictable) solution can be (temporarily) achieved.

Because Jung tends to highlight the ways in which this process occurs *within the individual psyche*, he also either ignores or plays down the involvement of the outer world (either in terms of the wider environment or in terms of relational connections with other humans or non‐humans). Simondon, on the other hand, puts great emphasis upon the notion of what he calls the *milieu*—a rich and suggestive notion that oscillates in meaning between ‘environment’ and ‘middle’, and thus destabilizes rigid divisions between inner and outer, intrapsychic and interpsychic. For Simondon, we need to acknowledge the collective as the milieu in which there can occur a “resolution of the tension between incompatible subjective problematics arising at the level of the lone subject” (Combes, 2013, p. 34). With the notion of transindividuation, Simondon articulates a reciprocal intersection between the two individuations—psychic and collective—which he describes as possessing a “systematic unity”. And exactly because transindividuation pushes beyond the individual into this collective direction, Simondon describes it as *psychosocial*.

It is important to acknowledge that Jung’s frequent emphasis upon individuality and interiority is in unresolved tension with those aspects of his psychology that implicitly highlight the importance of the collective:

[A] positive relationship between the individual and society or a group is essential, since no individual stands by himself, but depends upon symbiosis with a group. The self, the very centre of an individual, is of a conglomerate nature. It is, as it were, a group. It is a collectivity in itself and therefore always, when it works most positively, creates a group. ( Jung, 1973, p. 508)

It should also be emphasized that Jung, particularly in his later psychology, clearly seeks to establish the kind of transpersonal metaphysical base upon which a notion like the transindividual could be grounded. Concepts such as synchronicity and the psychoid support what Jung calls a “*unus mundus*” comprised not of separate atomized individuals but rather, a profoundly relational vision of wholeness.

As we have seen, the moments in which we break through to an awareness of this level of deep relatedness are also moments of affectivity. In other words, these moments when we are most aware of our connectedness are also the moments in which we are most affected and most affecting. The psychophysical soup we inhabit and make up is a soup of affect. 3

However, the fact is that this quasi‐psychosocial dimension of the life‐world is not systematically developed in Jung’s writings. Generally, his tendency to prioritize interiority over exteriority, combined with a persistent suspicion of groups or collectives, makes it all but impossible for Jung to enunciate a perspective that engages fully with the ultimate co‐inherence of interiority and exteriority, individual and collective.

As we have seen, when affect is perceived in individual/personal terms (as emotion) it tends to be interpreted by the subject or the observer in a relatively narrow way: my/your/his anger emerges out of my/your/his situation/predisposition/childhood. 4 This tendency to privatize affective events into the language of the individual ego seriously limits its relational meaning and simultaneously undermines its capacity to open up engagement with the psychosocial. However, as Simondon puts it, “affectivity is much more than just pleasure and pain; it is a way for the being at an instant to be situated according to a vaster becoming” (Simondon, 2020, p. 288). For Simondon the kind of relation contained by this ‘vaster becoming’ is one that breaks the bounds of the conventional person‐to‐person (ego‐to‐ego) relation. For Simondon, affect is a “continual link of the individual to itself and to the world, or rather the link between the relation of the individual to itself and the link of the individual to the world” (p. 273). In other words, affect knits together the way we relate to ourselves (the inner) and the way we relate to the world (the outer). Our sense of our own interiority is bound up, through affect, with our connection with the world, so that we can achieve knowledge of the former only through the latter, and vice versa.

However, because affect links our relation with ourselves to our relation with what Simondon calls the preindividual, we can also see it as a liaison between the personal and the archetypal (out of which future individuations will proceed). Simondon’s preindividual, just like Jung’s collective unconscious, challenges our narrow (ego)sense of ourselves. In Jung’s storm lantern dream in *Memories, Dreams, Reflections*, the little light he holds in the storm (his very individuality) casts a shadow that takes the form of a dark and threatening figure that follows him (Jung & Jaffé, 1989, pp. 88–89). The dream is saturated in the affective dimension of “terror” (p. 88). It is this affect that awakes Jung not only to knowledge but also to a new awareness of the crucial relation between his two personalities, and in effect between ego and self.

An affective event of this kind puts the ego itself into question. Because it poses “problems instead of resolving them” (Simondon, 2020, p. 277), affect disrupts the individual’s persistent tendency to detach and withdraw into its own encapsulated worldview. My secure sense of myself as a self‐possessed autonomous individual gets thrown into doubt by these affective relations that simultaneously link me to the pre‐individual (collective unconscious) and to the outer world. Simondon calls affectivity the “relational layer” that paradoxically “constitutes the centre of the individual” (p. 273).

Both Simondon and Jung agree that, in isolation, the ego doesn’t have the capacity to individuate. For Jung this is because the ego must undergo the painful challenge of a confrontation with the unconscious. Or as Jung puts it, the “experience of the Self is a defeat for the ego” (Jung, 1955, para. 778). Simondon agrees with this but goes further. For Simondon, the process of individuation is too big to be contained within any individual subject. Simondon’s transindividuation defeats not just the ego but the individual qua individual.

## Transindividuation

One way to grasp the process of transindividuation is to pay attention to the way it operates within and through the analytic encounter, via what we call transference‐countertransference dynamics. Although this is, psychosocially, a relatively narrow focus, it nonetheless provides us with an excellent opportunity to see how affect transcends the individual participants involved. As Nathan Field puts it, the sealed container of the analytic relationship gives us the chance to discern, in a specifically therapeutic setting, how “changes come about through the intensity of the feelings involved” (Field, 1996, p. 21). However, it is crucial to emphasize that affect operates in this way not only in analysis. These are universal dynamics which merely become easier to discern in the enclosed nature of the vessel and through the intensity of the alchemical heat generated.

Transference‐countertransference phenomena always possess what Jung describes as “an emotional and compulsory nature” (Jung, 1935, para. 316). In his late work on transference, Jung is particularly struck by the way these affective events extend beyond the separate psyches of either patient or analyst. This is why he focuses on what he calls the “bond” (Jung, 1946, para. 367) operating in the analytic vessel, and particularly on its powerfully affective nature. As he puts it, “the emotion of the projected contents always forms a link, a sort of dynamic relationship, between the subject and the object—and that is the transference” (Jung, 1935, para. 317). When describing this transpersonal field, he sometimes uses the term “the third” (when, for example, he says that “the two parties [are] involved in the transformation of the third and [are] themselves transformed in the process” [Jung, 1946, para. 399]).

Psychoanalysis, which in its earliest period remains in thrall to the Cartesian notion of the individual as a self‐reflexive subject, has tended to conceptualize the analytic event as at best a form of intersubjective communication between two separate self‐contained subject‐individuals. Nonetheless, in modern psychoanalysis a burgeoning awareness of the importance of transference and, particularly, countertransference phenomena (and a consequent emphasis upon the relational) has brought about important adjustments to Freud’s original approach. It is more or less assumed that as ego‐individuals we are invariably in intersubjective relation with other ego‐individuals and therefore that this relation is always already active in any analytic setting. However, even this *intersubjective* model of analysis—highlighting as it does the rich and complex relation between my (individual‐subjective) conscious and unconscious feelings about you and your (individual‐subjective) conscious and unconscious feelings about me—still ultimately relies upon the notion of the atomized individual, encountered, for example, in Winnicott’s “unit‐self” (Winnicott, 1992) and in Stern’s “core self” (Stern, 1998). For all its strengths then, the intersubjective lens we find in relational psychoanalysis tends to obscure precisely that phenomenon of thirdness that Jung considers so important.

As I have argued elsewhere, Jung’s psychology utilizes a model of the psyche that is radically different from that of psychoanalysis (Saban, 2016). This means that, despite Jung’s own personal bias toward interiority, analytical psychology’s wider emphasis upon the collective offers theoretical possibilities which provide post‐Jungians with a sensitivity to various connections, correspondences and communications that transcend narrow individualistic understandings of psychological existence. It is this that will help us identify the kind of psychic relation which can transcend that between person/individual and person/individual: that of affect operating within transindividual processes. No longer occurring within or belonging to either analyst or patient as separate individuals, affect emerges as a third, shared, collective relation.

## Analytic Affect

Post‐Jungian analyst and writer Nathan Schwartz‐Salant was a pioneer in extending and exploring what he called the interactive field—a reformulation of Jung’s “third”. For Schwartz‐Salant this interactive field is given priority over the individual subjects who occupy it. Significantly, he also highlights its affective character:

[O]ne never knows if an affect of fear, anger, hate or love comes from the analysand or from the analyst…. [S]uch emotions exist as a quality of the interactive field… a state in which the question of “whose contents” are being experienced cannot be determined. (Schwartz‐Salant, 1998, p. 24)

Alongside this *affective* dimension, Schwartz‐Salant also emphasizes the highly *relational* character of this field: It is, he says, “a realm in which relations per se are the main object, rather than the things related, such as complexes belonging to one or the other person” (Schwartz‐Salant, 1988, p. 50). In short, Schwartz‐Salant’s conceptualization of the interactive field provides us with an excellent lens through which to examine a clinical encounter with the autonomy of affect.

Unfortunately, post‐Jungian literature on transference‐countertransference has rarely thematized the role of affect within the interactive field. For example, Jacoby in his pioneering *The Analytic Encounter* (Jacoby, 1984), seems to be nodding in the direction of the interactive field when he mentions that a dream of a female patient “somehow… could have been a dream of my own in connection with the patient…. As a matter of fact, the dream really belonged to us both…” (Jacoby, 1984, p. 33). However, he follows up this observation with an extensive amplification of the complex symbolic meanings of the dream—they involve Bach, Mozart and the Magic Flute—and mentions the affective dimension of the field only in passing, noting that he was “truly moved” by the dream. Despite his recognition that the patient’s dream “belonged to [them] both”, his own emotional response to the dream is seen as his alone, useful merely because it “gives [him] an inner direction for the analysis”. Ultimately, the approach thus fails to move beyond the classical individualistic view of the relation.

In Schwartz‐Salant’s cases however we can see an increased openness to dwelling within the interactive field, and moreover a willingness to engage with the affective on its own terms, despite the inevitable confusions and difficulties it brings.

As a brief example, I intend to use a snapshot from one of Schwartz‐Salant’s cases, discussed in his 1998 book *Mystery of Human Relationship* (Schwartz‐Salant, 1998). I intentionally omit the biographical details of this case because I want to focus on the specifically *affective* dimension of the interactive field described and the light it sheds upon the transindividual.

Several years into Schwartz‐Salant’s work with a 50‐year‐old man, he has become increasingly aware of his patient’s rage (“a source of consternation for both of us” [Schwartz‐Salant, 1998, p. 89]) and begins to wonder about its destructive effect on their work together, noting his own tendency “to split off from focusing on [the patient]”. One day, Schwartz‐Salant asks his patient “if anger was attacking the connection between us”, to which the patient responds with a further question: “Whose anger?” Schwartz‐Salant admits that there is “no way to know” because, as he puts it, all that could be known is that “we were both in a kind of energy field in which anger was present”.

Note that affect here gets characterized as a quality of “the field” or the “third thing”, rather than as an emotion that belongs to either partner. As a result, what becomes constellated, we are told, is “a change in the quality of awareness of the texture and space around us” (Schwartz‐Salant, 1998, pp. 89–90). Interestingly, both participants feel, Schwartz‐Salant says, “as if an ‘other’ was present with us.”

In effect, their common experience of affect puts both partners into touch with something beyond the limits of their own separate individualities. Sometimes, Schwartz‐Salant notices, the participants feel as though they are “being inside and contained by it” but at other times it gets experienced as something “in the space between [them]” (p. 90). They are somehow the ‘subject’ of the field and somehow its ‘object’: it had the potential, for example, “to move us to behave in certain ways”.

How then should we understand this affective, interactive, autonomous “third” field of rage if, like Schwartz‐Salant we want to resist the temptation to describe it as occurring within or emanating from either of the individual participants? Schwartz‐Salant points here to the presence of a syzygic dimension of the phenomenon that reaches beyond the inner‐outer dichotomy. Both participants are simultaneously contained in, connected between, and confronted by an affective phenomenon whose autonomy pushes it beyond either of them as individuals.

From Simondon’s perspective, what the affect highlights is the pre‐individual relational field in which they meet. It is “more than individual” and so it shows up as outside the individual, but it also brings into play a larger, broader process that we can see as collective. In this case, the shared rage operates, according to Simondon, as a “link between the relation of the individual to itself and [a] link of the individual to the world” (Simondon, 2020, p. 273). Such a link is essential in transindividuational processes and goes to the heart of those transformational moments in analysis that transcend I and you, inner and outer.

Interestingly, at this point Schwartz‐Salant remarks that he “could easily have sidestepped this encounter” (Schwartz‐Salant, 1998, p. 90, thus highlighting the ease with which the uncanny nature of this phenomenon can be and often is avoided within analysis. Schwartz‐Salant himself offers an example of this from earlier in the analysis, at a point when massive fear enveloped both partners.

Asked by the patient “Where is this fear?” Schwartz‐Salant replied, “In both of us” and the patient then asked, “What could contain these feelings?” (Schwartz‐Salant, 1998, p. 87).

The patient’s anxiety is perfectly understandable in the face of an affective phenomenon that seems to be putting his very individuality into question. The thought behind it is: If I am sharing this fear with my analyst, is not the fear more powerful than either of us? And if so, what is to stop both of us becoming overwhelmed?

In “Psychology of the Transference”, Jung describes a patient who becomes so disturbed that he “is at a loss to know where his personality begins or ends” (Jung, 1946, para. 399). Jung’s patient is, he tells us, so perturbed that he “has to cling to the doctor as the last remaining shred of reality”. Jung continues, this

situation is difficult and distressing for both parties; often the doctor is in much the same position as the alchemist who no longer knew whether he was melting the mysterious amalgam in the crucible or whether he was the salamander glowing in the fire. ( Jung, 1946, para. 399)

It is precisely in these problematic circumstances, Jung says, that the “two parties… get involved in the transformation of the third and [are] themselves transformed in the process”. With this alchemical metaphor Jung conveys powerfully how the process puts into question the status of both patient and analyst as individuals—and is experienced as such. In the affective fire of the opus, Jung’s alchemist “no longer knows” if he is the subject or the object of what is happening, a striking parallel to Schwartz‐Salant’s case, in which both analyst and patient are somehow “inside and contained by” the affect which can also at times “move [them] to behave in certain ways”.

In such circumstances, there is a huge pull in the analyst to damp down the shared anxiety by assuming the role of, in Jacques Lacan’s words, “the one who knows” (1977, p. 230). In this case, confronted by the patient’s anxiety (“What could contain this fear?”), Schwartz‐Salant decides to provide a mythical‐alchemical interpretation of what is going on. What is required, he suggests to the patient, is to understand it as occurring within (contained by) the son‐lover myth (Schwartz‐Salant, 1998, p. 87). He justifies to himself this archetypal interpretation on the grounds that, as Jung says, “the archetype is the container”.

Strictly speaking, there is nothing ‘wrong’ with such an interpretation. Indeed, on a theoretical‐intellectual level it is probably correct. However, as Schwartz‐Salant rapidly realizes, in the light of the “interactive field”, he has taken a misstep. Seeking to “contain” the overwhelming fear‐anxiety (both his own and his patient’s) constellated by the mutual experience of transindividual affect he attempts to reassure the patient, and thereby reasserts the regime of individuality, thus shutting down the possibility of individuation. He relieves the terror of fragmentation and exerts control, but also short‐circuits the emergent transindividuational potential of the situation. 5

The ostensible goal of archetypal amplifications is to widen out the focus of the analysis from the individual‐personal toward the collective. Why then does such a move not open up a transindividuational dimension in this case? In practice, two factors work against it. First, the analyst’s evocation of a specific mythic‐archetypal pattern has the actual effect of bolstering his own authority (qua expert), and thus providing an escape from the mutuality of the shared affective experience. Second, by articulating what is occurring in the room in intellectual‐aesthetic terms, the analyst fatally undermines the affective character of the experience. All the creative potential that might have emerged out of a shared toleration of affect is lost. As Schwartz‐Salant ruefully remarks, although both partners now felt “more in control and far less fragmented … the experience between us was soulless, and embodiment in any depth was not possible” (Schwartz‐Salant, 1998, p. 88).

With this cautionary tale in mind, let us go back to where we were in Schwartz‐Salant’s case history. Here, an autonomous “third” field of affect has again arisen; a “high level of anxiety” is being experienced: a “degree of fear which pushed us to the edge of being contained” (Schwartz‐Salant, 1998, p. 90). We are again confronted with what feels like an urgent issue of containment.

On this occasion, however, Schwartz‐Salant responds differently. He now knows better than to sidestep the anxiety by invoking the kind of mythic or symbolic interpretation which might have “deconstructed and depotentiated the field between us in a particularly seductive way … [opting for] power in dealing with relationships over the experience of relatedness and suffering, all under the guise that it was for [the patient’s] own good” (Schwartz‐Salant, 1998, p. 90).

What emerges instead, once the rage had been acknowledged as somehow transcending both partners, is a level of toleration for the affect “without our knowing whose it was”. Subsequently, we are told, analyst and patient begin to “feel an energized sense of … body and its aliveness; and we became conscious of our bodies as energy fields” (Schwartz‐Salant, 1998, p. 90). At this point the nature of the field apparently changes again: “[The patient] felt that his body wanted to embrace mine, and I could also feel this sense of embrace, indeed, of a longing for him.” There are now “a pair of opposites defining our interactional space: rage and longing” (Schwartz‐Salant, 1998, p. 90).

We should note that Schwartz‐Salant is here pushing back against a general tendency within psychoanalytic, psychodynamic and Jungian traditions to regard what occurs in analysis as a problem for the individual, whether analyst or patient. In this case, for example, Schwartz‐Salant resists the temptation to “construct an interpretation” (Schwartz‐Salant, 1998, p. 87) which could, for example, have been based upon either his own feelings of abandonment in the face of his patient’s fragmentation or upon the patient’s childhood fearful relationship with his father. In both cases, the affective phenomenon might have been explained away as “a projective identification phenomenon” (Schwartz‐Salant, 1998, p. 87). Here too, the effect would have been to erase the transformative potential of this particular affective event, the essential significance of which was that it transcended the individual intrapsychic processes of either of the two participants. Such interpretations would be, Schwartz‐Salant suggests, “too limited and repressive of the field between us” (Schwartz‐Salant, 1998, p. 87). 6

Where then do these reflections leave Jung’s notion of the archetype as container? Does attention to the transformative nature of affect require us to abandon it? I think not. We need only to remember Jung’s insistence on the intensely affective nature of archetypal experience. Archetypes, he tells us, “are images and at the same time emotions. One can speak of an archetype only when these two aspects coincide” (Jung, 1961, para. 589). Crucially, Jung is emphasizing that the archetypal event requires neither an aesthetic recognition nor a cognitive understanding of the dynamics that emerge, but rather the affective undergoing of the archetypal as an experience of the Other. When an archetypal symbol spontaneously emerges out of a co‐individuational analytic process (for example, the synchronistic emergence of the scarab beetle in Jung’s famous account), it does indeed seem to possess the capacity to contain and simultaneously express something of what can otherwise feel like an overwhelming affective rawness. Here the symbol works to bridge affect and consciousness, without diminishing either.

However, this affective dimension of the archetypal experience occurs precisely when the interactive field becomes, in Schwartz‐Salant’s words, “the creative source of an interaction beyond the powers of the conscious personality”. In such cases the “discovery [is] accompanied by awe. This can be a numinous moment; it is a here and now experience of the archetypal transference” (Schwartz‐Salant, 1988, p. 49). What Jung describes as the numinous—the *mysterium tremendum et fascinans—*is itself a form of intense affect and one that pre‐eminently transcends the individual ego. As Rudolf Otto says, it is “felt as objective and outside the self” (Otto, 1923, p. 11). We might amplify the de‐centring potential of such a phenomenon by remembering the mythical episode of Actaion and Artemis. The hunter’s experience of coming across the goddess in the forest is profoundly decentring (i.e., dismembering). This transformative experience is precisely what the individual is required to undergo in the process of transindividuation. 7 Affect dis‐individuates the individual (as a formed entity) by forcing an encounter with what Simondon calls the not‐yet‐individuated pre‐individual: i.e., that share of the subject that is more‐than‐individual. Affectivity represents then a transformational mediation between this pre‐individual domain and the individual. Simondon enlists the notion of “metastability” when he describes this individuational transformation. The pre‐individual is dynamically metastable (neither stable nor unstable) in that it is made up of multiple tensions between potentials, waiting, as it were, to transform. This metastability can thus never be fully resolved but constantly triggers new individuations, which in turn bring about new metastabilities. To be metastable is to be always already on the brink of transformation, and out of it come new forms of collective and individual becoming.

Through a classical Jungian lens some of what I have discussed here could be understood in terms of the highly fissile energetic field that links and separates ego and Self. However, the transindividual enables us to re‐vision the concept of Self, such that it becomes an entity, or better a process, that is intrinsically and dynamically relational. Although Jung’s thought remains, to a certain extent, trapped in Cartesian understandings, his later psychology does make attempts to articulate transpersonal dimensions of psyche:

The self is relatedness.... The self appears in your deeds, and deeds always mean relationship; a deed is something that you produce which is practically outside of you, between yourself and your surroundings, between subject and object—there the self is visible. ( Jung, 1989, p. 795)

We should by no means think of this relatedness as a new‐age‐style cosiness of connection. It is rather that which opens one to a world of rawness and vulnerability in which the accustomed defences protecting the Cartesian “individual” have fallen away. It is in the face of this brute relatedness that Schwartz‐Salant’s patient asks, anxiously but understandably, “what could contain these feelings?”.

In fact, Simondon’s writings on anxiety shed useful light on such situations. For Simondon “anxiety is… not a passive experience; it is the effort made by a subject to resolve the experience of tension between preindividual and individuated within itself; an attempt to individuate all of the preindividual at once, as if to live it fully” (Combes, 2013, p. 32). Through a Jungian lens, this is the ego attempting to integrate or appropriate the Self by attempting to swallow it whole. For Simondon, however, this is not a problem that can be resolved solely on the intrapsychic level: it can only be tackled through an affective encounter with the transindividual.

Simondon’s approach sheds light on the ways in which therapy, as a means of dealing with individual neuroses, can itself become a neurotic process. When we approach the collective‐archetypal solely on the level of the individual (i.e., intrapsychically) then the true scale of the problem remains unaddressed. Like all unmet neuroses it simply shifts its ground and then reappears in a new setting. However, the analyst can avoid falling into this neurotic illusion by acknowledging the autonomy and power of the affect which, as we have seen, pervades the whole interactive field *and therefore transcends individuality*. Only in this way, by facilitating the transindividuational shift onto the level of a collective perspective, can the analyst avoid colluding in the patient’s neurotic ego belief that their anxiety can be relieved only by swallowing the self.

An understanding of neurosis as a *transindividual* problem—one that simply cannot be treated on an individual basis—makes a difference not only to the way we understand psyche but also to what it means to engage in psychotherapy. Analysis will always need to work with individual complexes and personal projections and analysts will always need to pay attention to those individual and personal issues that retain the capacity to distort and stunt individuation. Nonetheless, a transindivuational approach can help us see psychological life, and indeed life in general, as operating not only within the individual (however self‐aware) but rather within and through the affective dynamism of our relations—both with each other and with the world—and, more than that, within and through the relations between these relations. A renewed emphasis on affect might serve to divert attention from who (or what) is affecting or being affected by who (or what) and enable a focus onto the (transindividual) links or relations within which the affect operates. By refusing to prioritize either personal or collective, such a shift of focus opens new avenues for thinking the relational tension between the two. Such a re‐visioning also possesses the capacity to reveal new ways to think psychologically about the psychosocial, without falling into tired and well‐worn socio‐political tropes.

## Conclusion

In this paper, I have argued that a renewed emphasis upon affect can bring about an important shift within our psychological understanding. As a collective phenomenon that not only occurs within and between separate individuals but also transcends individual psychological processes, the notion of affect has the capacity to reveal relational connections that simply don’t show up within a conventional individualistic approach, mainly because they sit outside our usual narrow understandings of subjective or even inter‐subjective communication. By drawing a direct connection between the phenomenon of affect and Jung’s collective unconscious I have tried to give weight to Jung’s intuitions about the fundamentally collective and relational nature of human psychological life. The experiential immediacy of affect is what keeps us in touch with not only our bodies but with the world—the world out there and the world in here, both of which, it turns out, are in a state of intimate interplay.

I have focused here upon the ways affect shows up within transference and countertransference phenomena. Such experiences of transindividuation not only bring about individually realized personal insights or understandings, but more importantly they enable a transformative encounter with what Simondon calls “a vaster becoming”. The affective relations in which we all find ourselves—both those that hold us together and those that keep us apart—can thus be recognized as more fundamental than our status as separate individuals. A realization of this fact must inevitably alter the ways we understand not only the work of psychotherapy but also our place in the world.

## Footnotes

^1^
Jung seems to have derived it, at least in part, from the ideas of Eugen Bleuler, his boss at the Burghölzli. For Bleuler, affect is “the fundamental dynamic of the human psyche’s unity” since it functions as “the dynamic behind the thought and action process” (Vandermeersch, 1991, p. 80).

^2^
Jung became particularly aware of the importance of affect when working with patients suffering with Dementia Praecox or Schizophrenia back in 1908. Like Bleuler, Jung identified schizophrenia with a flattening of affective life. Not only do such patients find it hard to relate to others—making maintaining a functioning place in outer society very difficult—but the inner society of the psyche—the inner relations—are also disordered. It would seem that a minimal working level of affect is essential for the healthy functioning of psychological life.

^3^
This soup‐like dimension when encountered in analytic work problematizes but does not rule out the potential for analytic agency. As we will see in the clinical vignette discussed later, the analyst’s awareness of the soup‐like nature of the interactive field provides new possibilities for active engagement, albeit within a co‐individuational milieu that is more likely to evoke an attitude of Keatsian negative capability than a temptation to offer incisive interpretations.

^4^
Paradoxically, the highly personal character of emotion—and even the ways in which it shows up as personal—gets revealed in a form that is highly culturally modulated. My anger may be experienced by me as deep and personal, but the ways in which I can either express or even imagine my anger are highly specific to the culture in which I exist. We might suggest then that affect’s relation to emotion is analogous to the relation of archetype to archetypal image.

^5^
My intention here is not to dismiss those important analytic moments in which a symbol spontaneously emerges out of a co‐individuational analytic process. The naming (and even amplification) of the symbol in these cases does indeed seem to possess the capacity to contain, or at least to express, something of what can otherwise feel like overwhelming affective chaos. Here the symbol provides a bridge between affect and consciousness, without diminishing either. However, these events are quite different from those in which the analyst instrumentally utilizes a symbolic or archetypal frame with the intention of containing (or depotentiating) the raw affect that is emerging in the session.

^6^
Obviously, there are many occasions in analysis when such interpretations would be not only accurate but therapeutic. Schwartz‐Salant’s point is that there are also occasions, such as this, when they would be counter‐productive because they would undermine both analyst and patient’s capacity to fully experience the affective field.

^7^
My own mention here of the Actaion myth is a gesture in the direction of the affective‐archetypal. We can perhaps make some kind of (albeit second‐hand) affective contact with such an experience by reading, for example, Ovid’s account of the Actaion myth. However, it is in the Dionysian form of theatre, in which the myth is enacted in real time before an entranced audience, that this dismemberment shows up as an authentically collective‐affective experience.

## References

[joap13089-bib-0001] Alcaro A , Carta, S ., & Panksepp, J . (2017). The affective core of the self: A neuro‐archetypical perspective on the foundations of human (and animal) subjectivity. Frontiers of Psychology, 8, 1424. 10.3389/fpsyg.2017.01424 PMC5586212 28919868

[joap13089-bib-0002] Blackman, L. (2012). Immaterial bodies: Affect, embodiment, mediation. Sage.

[joap13089-bib-0003] Brennan, T. (2004). The transmission of affect. Cornell University Press.

[joap13089-bib-0004] Brooke, R. (1991). Psychic complexity and human existence: A phenomenological approach. Journal of Analytical Psychology, 36, 505–518.

[joap13089-bib-0005] Combes, M. (2013). Gilbert Simondon and the philosophy of the transindividual. MIT Press.

[joap13089-bib-0006] Clough, P. T. (2007). Introduction. In P. T. Clough & J. Halley (Eds.), The affective turn: Theorizing the social (pp. 1–33). Duke University Press.

[joap13089-bib-0007] Field, N. (1996). Breakdown and breakthrough. Routledge.

[joap13089-bib-0008] Jacoby, M. (1984). The analytic encounter: Transference and human relationship. Inner City Books.

[joap13089-bib-0009] Jung, C. G. (1907). The psychology of dementia praecox. *CW* 3 (paras. 1–316).

[joap13089-bib-0010] Jung, C. G. (1916). The transcendent function. *CW* 8 (paras. 131–193).

[joap13089-bib-0011] Jung, C. G. (1935). The Tavistock Lectures. *CW* 18 (paras. 1–415).

[joap13089-bib-0012] Jung, C. G. (1946). The psychology of the transference. *CW* 16 (paras. 353–539).

[joap13089-bib-0013] Jung, C. G. (1951). Aion: Researches into the phenomenology of the self. CW 9(ii).

[joap13089-bib-0014] Jung, C. G. (1954). Psychological aspects of the mother archetype. CW 9(i) (paras. 148–198).

[joap13089-bib-0015] Jung, C. G. (1955). Mysterium coniunctionis: An inquiry into the separation and synthesis of psychic opposites in alchemy . *CW* 14.

[joap13089-bib-0016] Jung, C. G. (1959). Good and evil in analytical psychology. CW 10 (paras. 858–868).

[joap13089-bib-0017] Jung, C. G. (1961). Symbols and the interpretation of dreams. CW 18 (paras. 416–607).

[joap13089-bib-0018] Jung, C. G. (1973). Letters, *Vol. 1: 1906–1950* (G. Adler & A. Jaffé, Eds., & R. F. C. Hull, Trans.). Princeton University Press.

[joap13089-bib-0019] Jung, C. G. (1989). *Nietzsche’s* Zarathustra. *C. G. Jung: The seminars. Vol. II, Part I and II. Notes of the seminar given in 1934–1939* , Ed. J. L. Jarrett. Routledge.

[joap13089-bib-0020] Jung, C. G. , & Jaffé, A. (1989). Memories, dreams, reflections. Vintage Books.

[joap13089-bib-0021] Lacan, J. (1977). Seminar of Jacques Lacan, Book XXIV, unpublished (Cormac Gallagher, Trans.).

[joap13089-bib-0022] Massumi, B. (2015). Politics of affect. Polity.

[joap13089-bib-0023] Otto, R. (1923). The idea of the holy: An inquiry into the non‐rational factor in the idea of the divine and its relation to the rational (J. W. Harvey, Trans.). Oxford University Press.

[joap13089-bib-0024] Pile, S. (2010). Emotions and affect in recent human geography. Transactions of the Institute of British Geographers, 35, 5–20.

[joap13089-bib-0025] Saban, M. (2016). Jung, Winnicott and the divided psyche. Journal of Analytical Psychology, 61(3), 329–349. 27192367 10.1111/1468-5922.12225

[joap13089-bib-0026] Saban, M. (2020). Simondon and Jung: Rethinking individuation. In C. Macmillan , R. Main & D. Henderson (Eds.), Holism possibilities and problems. Routledge.

[joap13089-bib-0027] Schwartz‐Salant, N. (1988). Archetypal foundations of projective identification. Journal of Analytical Psychology, 33(1), 39–64. 3350768 10.1111/j.1465-5922.1988.00039.x

[joap13089-bib-0028] Schwartz‐Salant, N. (1995). Introduction. In N. Schwartz‐Salant (Ed.), *C. G. Jung on alchemy*. Routledge.

[joap13089-bib-0029] Schwartz‐Salant, N. (1998). The mystery of human relationship. Routledge.

[joap13089-bib-0030] Simondon, G. (2020). Individuation in light of notions of form and information. University of Minnesota Press.

[joap13089-bib-0031] Slaby, J , & von Scheve, C . (2019). Affective societies: Key concepts. Routledge.

[joap13089-bib-0032] Stern, D. N. (1998). The interpersonal world of the infant: A view from psychoanalysis and developmental psychology. Karnac Books.

[joap13089-bib-0033] Vandermeersch, P. (1991). Unresolved questions in the Freud/Jung debate: On psychosis, sexual identity and religion. Leuven University Press.

[joap13089-bib-0034] Wetherell, M. (2012). Affect and emotion: A new social science understanding. Sage.

[joap13089-bib-0035] Winnicott, D. (1992). Review of Jung’s *Memories, dreams, reflections* . In R. Papadopoulos (Ed.), Carl Gustav Jung: Critical assessments. Routledge.

